# 多黏菌素B治疗粒细胞缺乏伴难治性革兰阴性菌血流感染27例临床观察

**DOI:** 10.3760/cma.j.issn.0253-2727.2023.06.007

**Published:** 2023-06

**Authors:** 萌 周, 慧珠 康, 骋圆 顾, 跃均 刘, 荧 王, 瞄 苗, 建红 付, 晓文 唐, 惠英 仇, 琤琤 傅, 正明 金, 彩霞 李, 苏宁 陈, 爱宁 孙, 德沛 吴, 悦 韩

**Affiliations:** 苏州大学附属第一医院，江苏省血液研究所，国家卫生健康委员会血栓及止血重点研究室，国家血液系统疾病临床医学研究中心，苏州 215006 The First Affiliated Hospital of Soochow University, Jiangsu Institute of Hematology, Key Laboratory of Thrombosis and Hemostasis of Ministry of Health, National Clinical Research Center for Hematologic Diseases, Suzhou 215006, China

**Keywords:** 多黏菌素B, 粒缺期, 难治性革兰阴性菌, 血流感染, Polymyxin B, Neutropenia, Refractory Gram-negative bacterium, Bloodstream infection

## Abstract

**目的:**

观察多黏菌素B治疗粒细胞缺乏（粒缺）伴难治性革兰阴性菌血流感染的疗效和安全性。

**方法:**

回顾性分析2021年8月至2022年7月在苏州大学附属第一医院血液科收治的粒缺伴难治性革兰阴性菌血流感染的病例，观察多黏菌素B治疗的疗效与安全性。

**结果:**

共纳入27例粒缺伴难治性革兰阴性菌血流感染的血液病患者，其中22例多黏菌素B治疗有效，首次发热至给予多黏菌素B的中位间隔为3（2，5）d；多黏菌素B的中位用药时间为7（5，11）d，中位退热时间为37（32，70）h。其中4例发生急性肾功能损伤，占14.8％，根据RIFLE标准，均为“损伤（Injury）”级别；色素沉着发生率为59.3％。

**结论:**

多黏菌素B治疗粒缺伴难治性革兰阴性菌血流感染具有较好的疗效和安全性。

## Abstract (en)

**Objective:**

To assess the efficacy and safety of polymyxin B in neutropenic patients with hematologic disorders who had refractory gram-negative bacterial bloodstream infection.

**Methods:**

From August 2021 to July 2022, we retrospectively analyzed neutropenic patients with refractory gram-negative bacterial bloodstream infection who were treated with polymyxin B in the Department of Hematology of the First Affiliated Hospital of the Soochow University between August 2021 to July 2022. The cumulative response rate was then computed.

**Results:**

The study included 27 neutropenic patients with refractory gram-negative bacterial bloodstream infections. Polymyxin B therapy was effective in 22 of 27 patients. The median time between the onset of fever and the delivery of polymyxin B was 3 days [interquartile range (*IQR*): 2–5]. The median duration of polymyxin B treatment was 7 days (*IQR*: 5–11). Polymyxin B therapy had a median antipyretic time of 37 h (*IQR*: 32–70). The incidence of acute renal dysfunction was 14.8％（four out of 27 cases）, all classified as “injury” according to RIFLE criteria. The incidence of hyperpigmentation was 59.3％.

**Conclusion:**

Polymyxin B is a viable treatment option for granulocytopenia patients with refractory gram-negative bacterial bloodstream infections.

粒细胞缺乏（粒缺）期感染是影响血液病患者治疗结局的主要危险因素之一[1]，其中革兰阴性菌引起的血流感染更是导致患者死亡的重要原因。近年来，随着各类抗生素使用的增加，多重耐药菌（multi-drug resistant, MDR）不断出现，使得粒缺期感染的管理变得更为困难。本文我们回顾性分析2021年8月至2022年7月在我科应用多黏菌素B治疗粒缺伴难治性革兰阴性菌血流感染的27例患者，以评估其疗效和安全性。旨在为粒缺期感染，尤其是MDR感染的治疗提供参考。

## 病例与方法

1. 病例：本研究为回顾性研究，纳入2021年8月至2022年7月收治于苏州大学附属第一医院血液内科的粒缺伴难治性革兰阴性菌感染且接受多黏菌素B治疗的患者。粒缺定义为中性粒细胞绝对计数（ANC）<0.5×10^9^/L，或预计48 h后ANC<0.5×10^9^/L。参考国内外对于难治性铜绿假单胞菌的定义[2]–[3]，难治性革兰阴性菌定义为对包含碳青霉烯或替加环素等高级抗生素的单药或联合抗感染方案治疗无效，或者药敏结果提示为MDR，即对3类或3类以上抗菌药物同时呈现耐药的细菌。入组标准：①接受过化疗或者造血干细胞移植预处理，处于粒缺期的患者；②单次口温≥38.3 °C（腋温≥38.0 °C）或≥38 °C（腋温≥37.7 °C）持续1 h，且排除药物或血制品输注等原因；③血培养或者宏基因组测序（mNGS）提示为革兰阴性菌感染，且满足难治性革兰阴性菌的定义。

2. 化疗及预处理方案：①化疗方案：地西他滨+IA（去甲氧柔红霉素+阿糖胞苷）；地西他滨+IAG（去甲氧柔红霉素+阿糖胞苷+G-CSF）；地西他滨+HAAG（高三尖杉酯碱+阿克拉霉素+阿糖胞苷+G-CSF）；维奈克拉+阿扎胞苷。②预处理方案：除多发性骨髓瘤患者应用BEAM方案（司莫司汀+依托泊苷+阿糖胞苷+美法仑），其他患者均采用改良BU/CY方案（司莫司汀+白消安+环磷酰胺+阿糖胞苷）进行预处理，HLA不全相合在BU/CY基础上加用抗胸腺细胞球蛋白（ATG）。

3. 抗感染用药方案：当明确为难治性革兰阴性菌感染时，立即启用多黏菌素B治疗，初始剂量为100 mg每12 h 1次（共2次），维持剂量为50 mg每12 h 1次，静脉滴注。根据《血液病/恶性肿瘤患者侵袭性真菌病的诊断标准与治疗原测（第六次修订版）》[4]以及《中国中性粒细胞缺乏伴发热患者抗菌药物临床应用指南（2020年版）》[1]进行联合用药方案的制定。体温正常后3 d开始降阶梯治疗或者停药。

4. 疗效判定：依照2004年《抗菌药物临床应用指导原则》对临床疗效进行判定。

5. 药物安全性判定：多黏菌素B治疗后的急性肾损伤应用RIFLE标准[5]进行评估，其他不良事件根据常见不良事件评价标准5.0（CTCAE 5.0）。

6. 统计学处理：应用R 4.1.0进行统计学分析。连续变量根据正态性与否分别运用*t*检验或秩和检验进行组间比较，分类变量则采用Fisher精确概率法进行组间比较，*P*<0.05为差异有统计学意义。

## 结果

1. 一般资料：27例接受多黏菌素B治疗的粒缺伴难治性革兰阴性菌感染血液病患者中，男19例，女8例，中位年龄42（35，57）岁。原发病包括：急性髓系白血病18例，急性淋巴细胞白血病4例，重型再生障碍性贫血、急性混合细胞白血病、浆细胞白血病、慢性髓性白血病（急变期）及慢性粒-单核细胞白血病各1例。27例患者中17例为化疗后出现粒缺，10例在造血干细胞移植过程中出现粒缺。

2. 病原学特征及药敏分析：27例革兰阴性菌感染患者中分离出铜绿假单胞菌株10株，其中MDR 2株，且均为碳青霉烯耐药菌株；肺炎克雷伯杆菌8株，MDR 3株，其中2株为碳青霉烯耐药菌株；嗜麦芽窄食单胞菌5株，其中MDR 1株，亦为碳青霉烯耐药菌株；大肠杆菌2株，其中MDR 1株（碳青霉烯耐药菌株）；阴沟肠杆菌和鲍曼不动杆菌各1株，均为MDR，其中阴沟肠杆菌为碳青霉烯耐药株。27例患者均存在革兰阴性菌血流感染的证据，其中22例通过外周血培养确认，5例通过外周血mNGS确认（分别为铜绿假单胞菌2例，嗜麦芽窄食单胞菌2例，大肠杆菌1例）。22株经血培养确诊者具体菌株和药敏见表1。除了明确的革兰阴性菌感染外，1例合并毛霉菌感染，1例合并巨细胞病毒血症。感染部位方面，27例患者中包含7例肺部感染，4例肠道感染，3例肛周感染，1例口腔感染，1例肝脾脓肿，1例中枢神经系统感染，1例混合感染（≥2处感染部位），9例感染部位不明确。

3. 多黏菌素B之前的临床抗感染方案：在抗感染方案调整为多黏菌素B前，19例（70.4％）患者应用碳青霉烯类药物，其中12例（44.4％）为亚胺培南，7例（25.9％）为美罗培南；5例（18.5％）为替加环素联合耐β内酰胺酶抑制剂（头孢哌酮舒巴坦或哌拉西林他唑巴坦）；3例（11.1％）应用头孢他啶阿维巴坦。

**表1:** 22株经血培养确诊的革兰阴性菌分布及耐药情况（株数）

菌种	株数	耐β内酰胺酶抑制剂	耐β内酰胺酶抑制剂复合制剂	耐氨基糖苷	耐氟喹诺酮	耐碳青霉烯
肺炎克雷伯杆菌	8	7	4	3	2	2
铜绿假单胞菌	8	8	4	2	2	2
嗜麦芽窄食单胞菌	3	3	1	1	2	1
大肠杆菌	1	1	1	1	1	1
阴沟肠杆菌	1	1	1	1	1	1
鲍曼不动杆菌	1	1	1	0	1	0

合计	22	21	12	8	9	7

4. 多黏菌素B单药/联合用药：本研究纳入的27例患者，有3例（11.1％）使用多黏菌素B单药抗感染治疗，16例（59.3％）应用含多黏菌素B的双药联合抗感染方案，其中3例联合β内酰胺酶抑制剂复合制剂（1例哌拉西林/他唑巴坦，2例头孢哌酮舒巴坦），4例联合氨基糖苷类（阿米卡星），3例联合碳青霉烯类抗生素（1例亚胺培南，2例美罗培南），2例联合替加环素，4例联合头孢他啶/阿维巴坦；余8例（29.6％）应用含有多黏菌素B的三药联合抗感染治疗，4例为联合替加环素以及碳青霉烯类抗生素（2例亚胺培南，2例美罗培南），2例为联合美罗培南和阿米卡星，1例为联合头孢他啶/阿维巴坦和阿米卡星，1例为联合替加环素和磷霉素。

5. 疗效分析：27例患者中，22例经过多黏菌素B的治疗后有效，用药后7、14、21、28 d累积有效率分别为 37.0％（95％ *CI* 21.9％～57.9％）、66.7％（95％*CI* 49.1％～83.2％）、77.8％（95％*CI* 61.0％～91.0％）、81.5％（95％*CI* 65.2％～93.3％）。粒缺期首次发热至多黏菌素B治疗的中位时间为3（2，5）d；多黏菌素B的中位用药时间为7（5，11）d，以上两者在多黏菌素B治疗有效组和无效组间分布差异均无统计学意义（*P*值分别为0.358和0.156）。多黏菌素B的中位退热时间为37（32，70）h。在27例患者中，共有5例出现了感染性休克，其中3例合并多器官功能障碍综合征（MODS）并最终发生死亡，从开始应用多黏菌素B到死亡的时间分别为2、5和12 d，死亡率为11.1％（95％ *CI* 0～22.2％）。根据《弥散性血管内凝血（DIC）诊断中国专家共识（2017年版）》，仅2例患者达到DIC的诊断标准，经过抗感染治疗后1～2天即脱离DIC的诊断。在多黏菌素B单药和联合用药疗效方面，单药多黏菌素B有效率为66.7％，联合用药有效率为83.3％，差异无统计学意义（*P*＝0.474）。此外，多黏菌素B在经血培养确诊组和经mNGS确诊组治疗有效率分别为86.4％和60.0％，差异亦无统计学意义（*P*＝0.464）。在感染部位方面，肺部感染的7例患者中，有6例经多黏菌素B治疗后好转，有效率为85.7％。

6. 安全性评估：根据RAFLE标准，27例患者中4例患者发生急性肾功能损伤（AKI），占14.8％，均为“损伤（Injury）”级别，出现肾功能损害的中位累积剂量为500（400，600）mg。有16例（59.3％）患者发生色素沉着，大部分患者在用药后2～3个月症状改善。1例患者出现神经毒性，表现为手脚轻度麻木，予对症治疗后好转。

## 讨论

据统计，在全世界范围内，四分之一至三分之一血流感染患者的病原菌为革兰阴性菌[6]；同时，由于革兰阴性菌生理结构特殊，极易变异成为耐药菌株，特别是MDR，其导致血流感染患者的死亡率超过30％[7]–[8]。在我国一项关于血液病患者的多中心前瞻性研究中，约有45％的粒缺合并发热是由革兰阴性菌引起的，其中经血流感染的比例更是超过了50％[9]。因此，针对血液病患者粒缺期合并革兰阴性菌（特别是MDR）的感染，如何制定有效的抗感染方案，依然是临床上亟待解决的问题。

多黏菌素是一种脂肽类抗生素，尽管历史上曾由于肾脏毒性被限制应用，但是目前仍然被认为是对碳青霉烯耐药的铜绿假单胞菌、肠杆菌以及鲍曼不动杆菌的最后一道防线[10]。临床使用的多黏菌素分为多黏菌素E（colistin）和多黏菌素B两种，前者以非激活的前体药物形式进入人体，而后者则是以活化的药物形式被人体摄入[11]–[13]，不过2017年的一项荟萃分析结果显示二者在针对多药耐药的革兰阴性菌疗效上并无明显差异[14]。

在本文报道的27例革兰阴性菌感染中，铜绿假单胞菌占总数的37.0％，肺炎克雷伯杆菌占29.6％，嗜麦芽窄食单胞菌占18.5％，鲍曼不动杆菌占3.7％，菌群分布情况与既往文献中粒缺血流感染细菌谱类似[9],[15]，其中部分菌群分布差异（如嗜麦芽窄食单胞菌的比例较既往报道偏高）可能是由于队列例数较低或者多黏菌素B用药前的抗感染方案差异所致。我国近期的流行病学调查结果显示，随着广谱抗生素的应用，除了铜绿假单胞菌对碳青霉烯耐药率略有下降以外，鲍曼不动杆菌、肺炎克雷伯杆菌以及肠杆菌属对碳青霉烯类药物的耐药率均呈现上升趋势[16]，而且近期发表的文献也显示，血液病合并耐碳青霉烯的革兰阴性菌（CRGN）感染是导致患者早期死亡的独立危险因素[17]，因此临床上对于CRGN感染应格外重视。在本队列经血培养确诊的病例中，共检出碳青霉烯耐药株7株，占总数的31.8％（7/22）；多黏菌素B治疗失败而发生死亡的3例中，感染的病原体分别为嗜麦芽窄食单胞菌、大肠杆菌和铜绿假单胞菌，均为碳青霉烯耐药株，故感染CRGN的死亡率为42.9％（3/7），提示尽管在多黏菌素B的治疗下，粒缺期合并CRGN感染仍然是临床抗感染治疗面临的一大挑战。

关于多黏菌素B的用药时机，原则上应该根据血培养的药敏结果进行调整，但是临床工作中往往受限于血培养阳性率较低、检测周期较长或者药敏结果与临床情况相左等因素，错过了最佳的用药窗口期。为了尽可能避免这种情况的发生，我们引入了难治性革兰阴性菌感染的概念，既保证了用药的科学性，也一定程度增加了临床的可操作性：当药敏结果尚未回报或者结果与临床用药相悖时，临床有证据显示现有包含碳青霉烯或者替加环素的抗感染方案对于患者疗效不佳，也可以尽早使用包含多黏菌素B的方案进行抗感染治疗。由于粒缺期这一特殊的免疫抑制状态，应尽早清除病原体，阻止感染的进一步发展，特别是CRGN的感染，多篇文献报道，感染性休克和高级生命支持的应用（机械通气等）是粒缺期合并CRGN感染早期死亡的独立危险因素[18]–[19]。此外，万理萍教授团队的研究显示粒缺期患者来源的大肠杆菌和肺炎克雷伯杆菌菌株产生广谱β-内酰胺酶的速率也比来自非粒缺期患者的菌株更高[15]，这提示我们尽快清除病原体也许可以避免更强耐药菌的产生，降低后续的治疗难度。我们汇报的病例队列中，多黏菌素B距离粒缺期首次发热的中位用药时间为3 d，中位退热时间为37 h，仅18.5％（5/27）的患者出现了感染性休克，说明早期判断难治性革兰阴性菌感染并进行针对性应用多黏菌素B可以改善患者的预后，为其争取后续的治疗机会。

由于上述血培养的缺陷，近年新兴的mNGS技术似乎一定程度可以弥补其不足。国外一项针对粒缺期伴发热的研究结果显示，以临床认可度为评判标准，患者外周血mNGS结果的敏感度、特异度、阳性预测值（PPV）和阴性预测值（NPV）分别为92％、70％、93％和63％[20]；国内付蓉教授团队的研究结果显示，血液病合并发热患者，以传统检查结果作为参照标准，外周血mNGS敏感性、特异性、PPV和NPV分别为75.68％、36.07％、41.79％和70.97％[21]。文章分析认为国内外mNGS检测效能的差异主要是由于外周血mNGS检出病毒阳性的标本缺乏有效的传统实验室检查为参照所致。所以现阶段指南建议尽管外周血mNGS阳性率高于传统血培养，但是血培养仍是疑似血流感染患者必做的检查，二者互为补充，但仍无法互相替代[22]。

关于多黏菌素B单药/联合用药的选择上，目前针对血液病合并粒缺期的患者主流的观点还是推荐联合用药。根据《中国中性粒细胞缺乏伴发热患者抗菌药物临床应用指南（2020年版）》，针对粒缺期合并碳青霉烯类耐药的肠杆菌科和铜绿假单胞菌、MDR/泛耐药/全耐药的铜绿假单胞菌或者鲍曼不动杆菌以及嗜麦芽窄食单胞菌，均推荐含有多黏菌素类药物的联合抗感染方案[1]。但是国际上仍有小部分学者认为多黏菌素联合其他抗生素并不优于单药治疗[23]–[25]。进一步分析后发现，虽然报道的总体有效率在单药和联合用药组无明显差异，但是联合用药组要么拥有更高或者更快的微生物清除率，要么合并较低的肾脏毒性，所以相比之下，联合用药组依然有其他方面的优势。此外，患者的基线数据（年龄、性别、基础病等）、微生物的种类、耐药性以及合并用药的种类均可能成为影响最终治疗结果的因素。在我们所汇报的27例患者中，分析发现单药多黏菌素B治疗组和联合用药组有效率并无明显差异，其中可能的原因还是纳入的病例数偏少。除了上述的因素以外，感染的部位和给药方式也会影响治疗的效果。血液病合并粒缺发热的患者最常见的为肺部感染[9]，但是又由于肺部结构的特殊性，静脉用药难以维持组织中的药物浓度，因此相关指南建议可以在全身用药的基础上加用吸入性抗生素。本文中的27例患者，共有7例为肺部感染，占25.9％，较文献汇报比例偏低，这7例中多黏菌素B均为静脉给药，有效率可以达到85.7％（6/7），这可能与肺部感染病灶范围较小以及多黏菌素B的及时用药有关。因此，在粒缺期合并难治性革兰阴性菌感染的治疗中，还是较为推荐多黏菌素B联合用药方案，至于联合用药的选择需要综合考虑病原菌的种类、感染的部位、患者的耐受性等，相关的证据也需要通过更严谨的随机对照试验来获得。

不良反应方面，多黏菌素B主要有肾毒性、神经毒性和皮肤色素沉着。其中临床最为关注的就是用药相关AKI，文献报道多黏菌素B相关AKI的发生概率不一，波动在7.1％～54％[26]–[35]。绝大多数研究都依据RIFLE标准来评估肾功能的变化，少数研究仅以肌酐较基线的升高倍数来评估（缺乏尿量等临床症状评估），这可能是导致AKI发生率差异较大的原因之一；另外合并用药以及感染的严重程度等，也会影响到AKI的诊断和分级。有学者认为，多黏菌素B应用剂量的增加会降低感染相关的死亡率，但是也会显著增加肾功能的损害，然而这个界值在文献中也不甚统一，一般认为超过150 mg/d就会导致严重的肾功能损害[26],[30]。本研究中，总体AKI发生率为14.8％，与历史文献对照处于相对较低水平，分析原因可能与用药剂量较小（有的中心采用150 mg/d甚至更高）以及起效后及时停药有关。

本研究仍然存在一定的不足：①本质为回顾性分析，且纳入病例偏少，证据级别偏低；②为单臂描述性研究，缺乏对照组。

本研究报道了多黏菌素B在血液病患者粒缺期合并难治性革兰阴性菌血流感染中的治疗情况，一定程度上反映了多黏菌素B在常规剂量下拥有较好的疗效和较少的不良反应，但是未来仍然需要严谨的前瞻性随机对照临床试验来进一步验证多黏菌素B在血液病合并感染领域的有效性和安全性。
